# Crosstalk between cancer and immune cells: Role of tumor‐associated macrophages in the tumor microenvironment

**DOI:** 10.1002/cam4.2327

**Published:** 2019-06-20

**Authors:** Jing Wang, Danyang Li, Huaixing Cang, Bo Guo

**Affiliations:** ^1^ School of Life Sciences Northwestern Polytechnical University Xi'an China; ^2^ Institute of Pharmaceutical Science King's College London London UK; ^3^ Department of Ophthalmology West China Hospital of Sichuan University Chengdu China

**Keywords:** angiogenesis, chronic inflammation, immune suppression, TAMs, tumor

## Abstract

Tumor microenvironment is a complex system that contains multiple cells and cytokines. Among the multiple immune cells, macrophage is particularly abundant and plays an important role throughout the tumor progression process, namely, tumor‐associated macrophage (TAM) in this special tumor microenvironment. Many kinds of cytokines from TAMs and other immune cells in tumor niche are involved in the linkage of inflammation, immunity and tumorigenesis. Inflammatory responses induced by TAMs are crucial to tumor development of different stages. This review highlights the critical role of TAMs in the linkage of inflammation, immunity, and cancer. It outlines the molecules of inflammatory cytokines, chemokines, and growth factors mainly from TAMs in tumor microenvironment and their functions in tumor development during the major issues of angiogenesis, chronic inflammation, and immune suppression. Additionally, the signaling pathways involved in tumor progression and the crosstalk between them are also summarized.

## 1 INTRODUCTION

The immune‐associated cells in tumor microenvironment are multitudinous, mainly including macrophages, dendritic cells (DCs), myeloid‐derived suppressor cells (MDSCs), T cells, mast cells, and natural killer (NK) cells. All of them play critical roles in the resistance to infection and other diseases.^1^ Recent evidences strongly support the opinion that immune system has both positive and negative effects on tumorigenesis, and the inflammatory microenvironment is an essential component for tumors.^2^, ^3^, ^4^, ^5^ On one hand, the innate immune response can protect host from virus‐induced tumor by inhibiting or eliminating viral infection; on the other hand, the rapid removal of pathogens and inhibition of inflammation will construct a suitable inflammatory microenvironment for tumor formation. However, the diverse role of inflammation in tumor development and other diseases largely depends on cytokines secretion and the diverse interaction with its neighbors. Indeed, there is a complex interaction and balance between inflammation, immunity, and tumorigenesis.

In tumor microenvironment, the role of inflammation is type and level dependent. The acute inflammation is known as the defense of normal host against infection and injury, as well as extirpate tumor cells. But an excessive and uncontrolled inflammation will trigger the chronic inflammation, which would destroy the host immunity and increase the risk of tumorigenesis.^6^, ^7^ Many evidences have confirmed that the chronic inflammation make a huge contribution to tumorigenesis, but the underlying molecular mechanism is intricate and still unclear.

Several intermediate processes including angiogenesis, chronic inflammation, and immune suppression may account synergistically for tumor development and constraining. In chronic inflammation process, many molecules including inflammatory cytokines, chemokines, growth factors, reactive oxygen and nitrogen species are involved which could trigger the tumor angiogenesis, DNA damage, gene mutation, as well as the battle between immunosuppression and promotion. Additionally, tumor‐associated macrophages (TAMs) are of particular importance in the linkage between inflammation and cancer.

In this paper, we will discuss the function of tumor‐related immune cells, mainly focusing on the role of TAMs and the cytokines in tumorigenesis. The involved signaling pathways in correlation of tumor development with inflammation will be profiled simply. This review tries to provide the latest progress in the field of inflammation‐associated tumor and brings more profound ideas to researches.

## 2 IMMUNITY IN CANCER

### 2.1 Tumor‐related immune/inflammatory cells

Tumor is a product of the complex interaction between malignant cells and other normal cells (such as immune cells) from a single initiated cell into a fully tumor. Immune cells in tumor microenvironment mainly include TAMs, DCs, T cells, NK cells, MDSCs and mast cells, which play diverse roles in tumor procession stages.^1^

The conventional opinions hold that TAMs are one of the M2‐like macrophages due to its high expression of antiinflammatory marker genes, such as interleukin‐10 (IL‐10) and IL‐1 receptor alpha (IL‐1Ra), which make a huge contribution to tumor growth and the subsequent development. Moreover, the primary TAMs can recruit monocytes to tumor site by secreting chemotactic factors of CCL2, CCL5, CCL7, CXCL8 and CXCL12 which can be polarized to M2‐like phenotype with the stimulation of IL‐4, IL‐6, IL‐10, IL‐13 and transforming growth factor‐beta (TGF‐β).^8^, ^9^ Besides, products of tumor‐promoting growth factors from TAMs, such as epidermal growth factor (EGF), also make efforts to neovascularization and modulating immune response. In this process, the matrix metalloproteinase (MMPs) synthesis which have a significant impact on angiogenesis is regulated by vascular endothelial growth factor (VEGF), platelet derived growth factor (PDGF), fibroblast growth factor (FGF), and TGF‐β.^10^, ^11^, ^12^ As reported recently, TAMs have a special transition period from M1‐like to M2‐like phenotype, which means they are not just belonging to M1‐like or M2‐like phenotype at the whole process of tumor progression. At the early stage of tumor initiation, TAMs are M1‐like phenotypes before transferring to the M2‐like type. Besides, it is requisite to point out that M2‐like macrophages are further sub‐divided into 4 subtypes, namely, M2 a, b, c, and d.^13^ And the markers of different subtypes are different. In this paper, only the specific markers for M1‐like and M2‐like (for all subtypes) macrophages were concluded in Table 1.

**Table 1:** Specific markers for M1‐like and all subtypes M2‐like macrophages

Cell type	Recognized markers	Reference
M1‐like macrophages	CD80^+^, CD86^+^, TNF‐α, VEGF, SOCS3, CCR7,	14, 15, 16, 17, 18, 19
M2‐like macrophages	CD163, IL‐10, SOSC1/2, CD206, CCL‐18, PDGF‐BB, MMP	15, 19, 20, 21, 22, 23, 24

DCs are a kind of marrow‐derived cells that distributed in almost all tissues. They build a communication between innate immunity and adaptive immunity, and plays a critical role in the specific immunity.^25^, ^26^ They are also an important component in various tumor microenvironments and regulated significantly by IL‐10, VEGF and prostaglandin E2 (PGE2).^27^, ^28^, ^29^, ^30^ In general, the role of DCs in tumor development is controversially. Previous in vivo and in vitro researches have indicated that DCs could not only promote tumor cells survival and colony formation, but also showed a promising ability to induce antitumor immunity.^31^, ^32^ Besides, DCs‐derived exosomes can also initiate the antitumor process via activating T cells and NK cells.

MDSCs are another type of host immune cells in tumor microenvironment that include two major subpopulations of monocytic and granulocytic MDSCs.^33^ They were defined by the expression of plasma membrane markers and the content of immune suppressive molecules. The proliferation of MDSCs is mainly mediated by growth factors, cytokines, and MMPs.^34^ They coordinated with other cells to regulate immune response by triggering the immune‐suppression and promoting antiinflammatory phenotypes.^33^, ^35^, ^36^ The immune‐suppression of MDSCs is determined by cytokines of IL‐4, IL‐13, TGF‐β and interferon‐γ (IFN‐γ), thus to synthesis inducible Nitric Oxide Synthase and arginase. Moreover, MDSCs also promote angiogenesis, which can improve nutrients and oxygen transmission that are favorable for subsequent tumor growth and metastasis.^37^ Recently, MDSCs as well as its microenvironment are considered as promising candidates to promote tumor cells damaged and clearance.

T cells are a kind of killer to tumor cells, but they still cannot control tumor development due to the properties of low activity, exhaustion, and aging.^38^ The immunity of T cells is suppressed by cytokines, chemokines, and enzymes from TAMs in tumor microenvironment via different signaling pathways. The interactions between T cells and TAMs can be used to interpret the relationship between immunity and inflammation partially.

NK cells are a kind of innate immune cells in tumor microenvironment. The killing activity of NK cells is increased significantly by the inflammatory cytokines of tumor necrosis factor alpha (TNF‐α), IFN‐α, IL‐12, and other ILs. However, the proliferation and inherent functions of NK cells are usually regulated by the secreted cytokines of PGE and TGF‐β through autocrine and paracrine manners or inhibited via contacting with other cells.^39^

Mast cells, derived from bone marrow hematopoietic progenitor cells, are usually involved in the biological processes of tissue remodeling, wound healing, and angiogenesis.^40^ Recent evidences have confirmed that the mast cells also participate in tumor cells’ proliferation and metastasis by releasing mediators which involved in remodeling tumor microenvironment.^41^ They promote the inflammatory reactions by producing proinflammatory factors,^42^ and also modulate the immune responses by hydrolyzing chemokines and cytokines.^43^ In tumor microenvironment, the amount of inflammatory factors, immune‐suppressive factors and chemokines from mast cells are helpful to establish an inflammatory and immunosuppressive condition.^44^

### 2.2 Relationship between inflammation and tumor

Previous researches have found that the inflammation may be an inducer for tumor initiation. Macrophages, almost distribute in all tissues, are a major kind of immune cells in tumor microenvironment that regulate the inflammation. Generally, two broad phenotypes of M1‐like and M2‐like are involved. The type of M1‐like, which was induced by TNF‐α, granulocyte‐macrophage colony‐stimulating factor (GM‐CSF) and IFN‐γ, is defined as proinflammatory cells due to several special characteristics. The contributions of M1‐like macrophages to tumor development are double‐edged. The viruses and bacteria in cells can be killed by M1‐like macrophages via secreting inflammatory factors of IL‐12 and IL‐23. While it also enhances the metastatic potential of cancer cells via activating nuclear factor‐κB (NF‐κB) signals.^45^ M2‐like macrophages, which were induced by IL‐4, IL‐13 and glucocorticoids, have been found to involve in biological processes of angiogenesis, tissue remolding, wound healing, and antiinflammation.^46^, ^47^ More importantly, M2‐like type can specially promote angiogenesis which facilitates tumor growth and metastasis due to the favorable nutrient and oxygen transportation, and tissue remolding and immunosuppression as well.^48^ The angiogenic potential is enhanced through generating high cyclooxygenase‐2 expression, resulting in the elevated release of VEGF and FGF from both TAMs and tumor cells.^49^ CD163, a specific important marker for M2‐like macrophages, was initially found to be expressed in perivascular macrophages, but not in parenchymal microglia by Borda et al.^50^ It is also worth mentioning that CD163 is involved in protumoral activation of macrophages and subsequent development and progression of tumors in mice and humans, but the expression pattern is closely associated with the species in recent report.^51^ Taken together, it is difficult and complex to distinguish the macrophage phenotype due to a persistent switch between M1‐like and M2‐like that there is some overlap between two phenotypes.

In vivo, the sustained activation of antioncogene caused by gene mutation can induce the production of inflammatory mediators such as leukocyte aggregation, which makes contributions to oncogenesis. However, the inflammatory microenvironment induced by bacterial infection also increases the cancer risk. In general, TAMs are considered as one of the most influential factors that account for inflammation‐associated tumor development. The balance between antineoplastic immune and oncogenic inflammation is so intricate that not only depend on immune cells and mesenchymal cells activation, but also strongly related to the pivotal cytokines, chemokines, growth factors etc.^52^, ^53^, ^54^ Some researchers hold the opinion that the oncogenesis induced by majority cytokines belongs to the M1‐like of proinflammatory type. For example, M1‐like macrophage‐derived TNF‐α can promote reactive oxygen species (ROS) accumulation in latent tumor cells that can damage various proto‐oncogenes and antioncogenes, such as p53.^55^ Moreover, the EGF and IL‐6 induced STAT3 (signal transduction and transcription activator 3) activation will finally result in tumor formation.^56^ In contrast, others demonstrated that large amount of proinflammatory molecules from M1‐like macrophages are involved in killing cancer cells.^57^

Generally, it is well recognized that TAMs show the M1‐like characteristic properties at the early stage of tumor formation, but more like specific M2‐like phenotype in tumor progress and then support tumor growth by promoting angiogenesis, remodeling matrix, and secreting antiinflammatory cytokines.^48^ The increased secretion of IL‐6 and IL‐10 from TAMs is helpful to construct a tumor microenvironment with a feature of immune suppression, angiogenesis, and antiapoptotic.^58^, ^59^ Furthermore, the redundant cytokines and chemokines in this niche will attract more macrophages and other inflammatory cells to tumor location, then generate more cytokines accumulation and thus forming a circle.^11^ In addition, M2‐like macrophages are effective in many steps of tumor development due to the specific properties which is necessary for tumor development including angiogenesis, invading tissue, remodel matrix, enough growth factors, chronic inflammation, and infinite proliferation.^60^, ^61^

### 2.3 Role of TAMs in tumor

In general, three main factors are involved in tumor development including tumor angiogenesis, tumor invasion chronic inflammation, and immune suppression, respectively. The role of TAMs in these biological processes was discussed briefly in the following sections.

#### 2.3.1 TAMs in tumor angiogenesis

##### Angiogenesis in tumor

Tumor growth and metastasis depend largely on the angiogenesis, which is the growth of new blood vessels from existing ones that surrounded by the growing tumor mass. When vessel is absent, the tumor cell colony is supplied with sufficient nutrients and oxygen from surrounding environment by means of diffusion, but only limited to small mass. By the time, tumor will become dormant and degenerate gradually in the areas that lack of blood vessels. Otherwise, tumor will grow rapidly and transfer to other sites once attached to new vessels that contain enough nutrition.^62^ In fact, the interaction between tumor growth and angiogenesis is mutual. As mentioned previously, angiogenesis is a critical step for tumor growth by providing nutrition in this process. On the other hand, the local endothelial cells in tumor tissue microenvironment will proliferate rapidly to form vascular buds by the stimulation of growth factors from both TAMs and cancer cells firstly. Subsequently, the vascular buds will grow towards tumor and then secrete growth factors. The growth of vascular buds in tumors can then irritate the tumor again by generating factors that stimulate angiogenesis, thus forming a circulation. Besides, the endothelial cells can also accelerate tumor growth and progression by promoting angiogenesis.^63^ In conclusion, the angiogenesis process lays a foundation for tumor metastasis in the feedback regulating network between angiogenesis, endothelial amplification, and tumor progression. It not only increases the opportunities of tumor cells entering the blood vessels, but also provides a place for the combination of tumor cells and matrix that is necessary for metastasis.

##### TAMs in angiogenesis

Besides hypoxia, inflammation is also a critical factor that accounts for tumor angiogenesis. The angiogenesis in tumor is initiated by budding epithelial cells on original vascular system which are different from in embryonic development. In tumor microenvironment, blood vessels are twisted or swollen rather than normal state. To proliferation, the activation of VEGF and other growth factors is essential for the differentiation of silent endothelial cells toward angiogenesis forms.^64^ Recently, numerous studies have demonstrated that TAMs function as major producers of proangiogenic factors in malignant tumors.^65^, ^66^ It is closely associated with the productions of IL‐1β, VEGF, TGF‐β, α and other cytokines showing the stimulations on other cells in tumor stroma and then create a suitable microenvironment for angiogenesis.^67^, ^68^, ^69^ VEGF is known as a major proangiogenic cytokine released from TAMs in several types of cancer. It is also released by cancer cells under the inducer of IL‐1β from TAMs. TGF‐β from TAMs can also give rise to VEGF expression via an autocrine effect. Moreover, MMP‐9 and other protease from TAMs have a strong modulation on extracellular matrix (ECM) in the budding process, whose proteolytic function will support the activated endothelial cell invasion and other cells migration in this period.^70^, ^71^ The elevated expression of MMP‐9 by TAMs also mediates the release of bioactive VEGF. In conclusion, the involvement of TAMs in tumor angiogenesis not only limites to the angiogenic factors in tumor cells, but also makes a contribution to the activation of proangiogenic procedure in tumor cells by liberating cytokines from TAMs.

#### 2.3.2 TAMs in tissue chronic inflammation

##### Chronic inflammation in tumor

In normal tissue remolding, chronic inflammation is terminated when body receives a signal that the repair has been completed. However, in the tissues with carcinogenic mutations, the initiation of tumor growth is often regarded as an uncontrollable repair response that activated by the tissue injury. Therefore, cancer cells cannot respond correctly as the normal cells to repair response. As a result, the repair process will be continuously activated due to the incorrect damage has always been there that leads to a sustained chronic inflammation. Chronic inflammation can also be resulted from other possibilities of obesity, environmental exposure, and infections. Additionally, cell aging and the accumulation of damaged DNA can also cause tumor‐promoted chronic inflammation.^72^ Chronic inflammation which occurs prior to tumor will give rise to its development by promoting angiogenesis, inducing local immune suppression and oncogemic mutations. Moreover, a lot of cytokines and growth factors, which facilitate angiogenesis and support tumor growth and metastasis, will be produced during chronic inflammation in tissue repair. More importantly, micro‐RNA (a small noncoding RNA molecule which functions in RNA silencing and posttranscriptional regulation of gene expression) will be changed by the inflammatory signals of IL‐6 and NF‐κB in chronic inflammation, thus blocking the cancer cells apoptosis and increasing the potential of infiltration and metastasis.^73^ Besides, the enhanced production of inflammatory factors during chronic inflammation will be amplified and developed to tissue infection that increases the risk of tumorigenesis.^74^ Therefore, a persistent chronic inflammation should be maintained in the presence of TAMs which will support tumor progression.

##### TAMs in chronic inflammation

There are many differences between chronic and acute inflammation. Chronic inflammation is caused by the persistent inflammatory stimulation and identified by the existence of macrophages and monocytes, as well as the growth of connective tissue and vessels. Macrophages, are considered as the most important inflammatory cells in chronic inflammation mainly ascribed to the cytokines it produced, including chemokines, growth factors, acid compounds, and other characteristic metabolites. In tumor niche, the presence of TAMs can maintain a chronic inflammation by releasing inflammatory molecules that initiate tissue remolding process.^75^ Besides, the growth factors of VEGF, PDGF, TGF‐β, and FGF from TAMs in chronic inflammation process also play important roles in supporting vascular ingrowth which accounts for tumor development.

#### 2.3.3 TAMs in immune suppression

##### Immune suppression in tumor

A normal immune system is necessary for the control of malignant disease. Similarly, cancer‐related immune suppression makes a huge contribution to its development. In tumor microenvironment, the significant relationship between immune response and oncogenesis is connected by the inflammatory cytokines‐mediated immune activation and impairment. Cytokines from TAMs and other leukocytes always compete with the immune inhibiting molecules which can cause damage to the immune system. Moreover, the inflammation‐associated immune suppression localized in tumor site has a vast of effects on other activities of immune cells, including DCs, T cells and NK cells as discussed previously. Eventually, the incompetent immune system will not make efforts to the biological events that against tumor.

##### TAMs in immune suppression

Another important role of TAMs in protumorigenic process is the suppression of antitumor immune responses. In current opinion, TAMs are tending to be M2‐like phenotypic due to the production of IL‐10, TGF‐β, and PGE2, which are typical markers of M2 type that promote tumor angiogenesis and tissue remodeling.^76^ In tumor microenvironment, many other cytokines are liberated from TAMs to promote tumor invasion, such as M‐CSF, MMPs, and EGF. Interestingly, the secretion of M‐CSF can cause TAMs to maintain the M2‐like phenotype, thus to form a circulation that promote tumor development continuously. Besides, the chemokines released from TAMs can attract other cells (eg Th2 cells and regulatory T cells) to tumor niche and then construct an immunosuppressive environment. For example, TAMs can secrete various cytokines, chemokines, and enzymes that can suppress T cells activity by recruiting the natural regulatory T cells or depleting l‐arginine into the tumor microenvironment.^38^ Numbers of researches have suggested that TAMs with M2‐like phenotype, who express a number of cytokines and decoy receptor molecules (eg IL‐10, CCL‐18, PGE2, TGF‐β, dIL‐1R, and Eotaxin‐2/CCL24), are immune suppressors and facilitate angiogenesis and tumor development.^77^, ^78^, ^79^, ^80^ The PGE2, TGF‐β and other chemokines in tumor microenvironment can also hinder the maturation of DCs, which is breaking the balance between innate immunity and adaptive immunity, as well as suppressing the activity of T cells and NK cells.^26^, ^81^ In a recent study, siglec‐15 which was found to be abundant in macrophages but lack in other immune cells or normal human tissues, was suggested to be a macrophage‐associated suppressive molecules to T cells. It was also proposed by the in vivo experiment that the siglec‐15 deficient mice was resistive to tumor growth by promoting the responses of T cells,^82^ and siglec‐15 is recommended to be a promising target for normalized cancer immunotherapy.^83^ programmed cell death ligand 1 (PD‐L1), a ligand for T‐cell inhibitory receptor PD‐1, was suggested to be an active agent in the immune suppression. It was confirmed that PD‐1 can make contributions to immune suppression from both tumor and host.^84^ The in vivo study focused on the mechanism of how the ligand for PD‐1 regulates antitumor immunity revealed that the TAM‐derived PD‐L1 contributes predominantly to suppress antitumor immunity than the host‐derived one. Further investigation indicated the importance of TAM‐expressed PD‐L2, another ligand for PD‐1, in the suppression of antitumor immunity.^85^ In fact, the immunosuppressive activity of TAMs is largely depended on the cytokines liberation that acts on T cells and the subsets.

**Figure 1:**
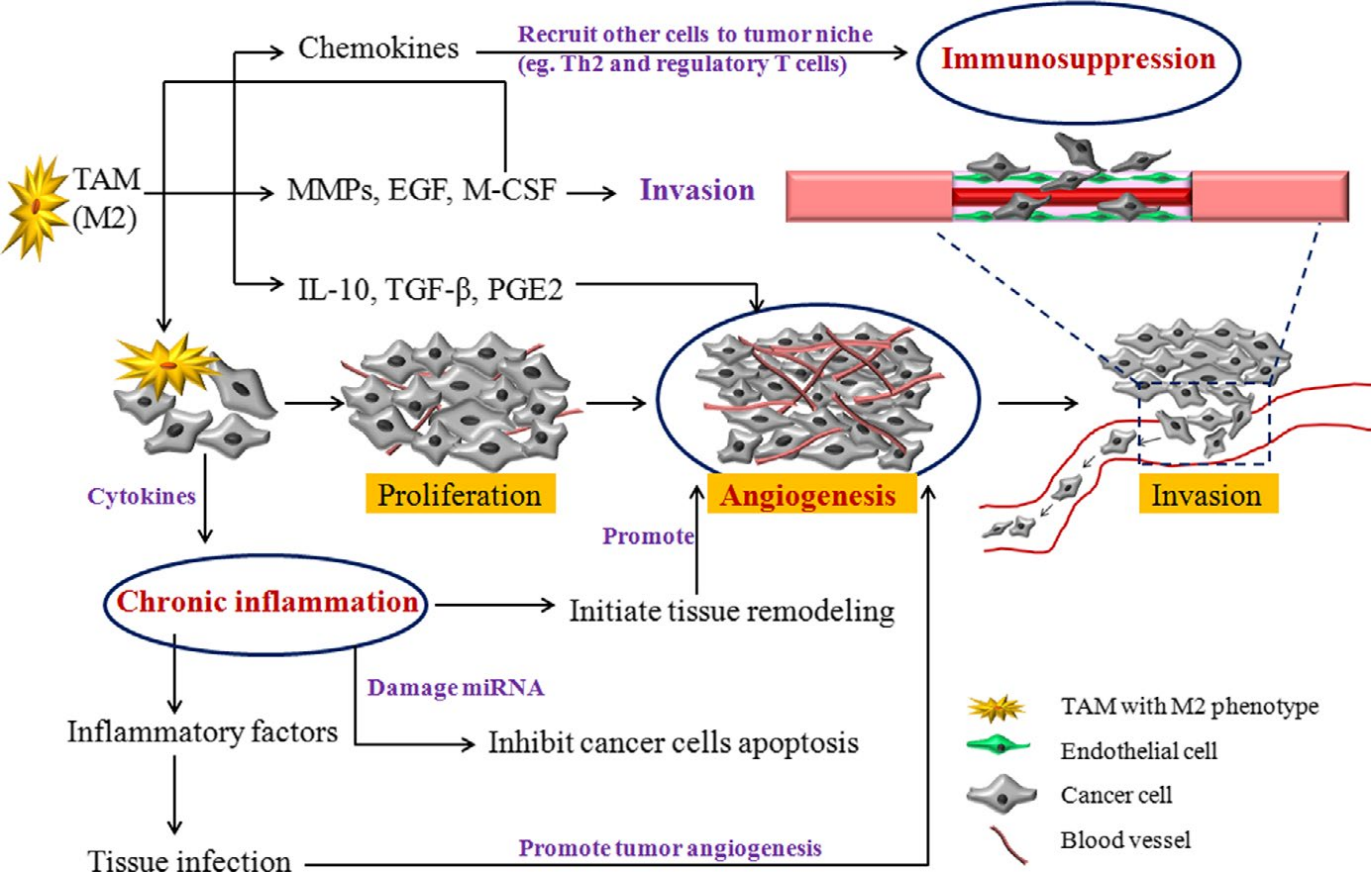
The role of TAM in tumor development via the cooperation of angiogenesis, chronic inflammation and immune suppression. Abbreviations: EGF, epidermal growth factor; IL‐10, interleukin‐10; M‐CSF, macrophage colony‐stimulating factor; MMP, matrix metalloproteinase; PGE2, prostaglandin E2; TAM, tumor‐associated macrophage; TGF‐β, transforming growth factor‐beta

In conclusion, TAMs make a huge contribution to tumor development by the cooperation of angiogenesis, chronic inflammation, and immune suppression as simply profiled in Figure 1.

### 2.4 Inflammation and cytokine‐involved signaling pathways in tumor

#### 2.4.1 Inflammatory tumor microenvironment

**Figure 2:**
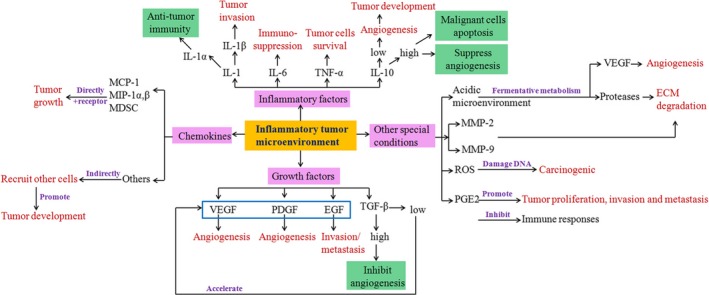
The main cytokines and special conditions in tumor microenvironment. Abbreviations: ECM, extracellular matrix; EGF, epidermal growth factor; IL, interleukin; MCP‐1, monocyte chemotactic protein‐1; MDSC, myeloid‐derived suppressor cell; MIP‐1α,β, macrophage inflammatory protein‐1α,β; MMP, matrix metalloproteinase; PDGF, platelet derived growth factor; PGE2, prostaglandin E2; ROS, reactive oxygen species; TGF, transforming growth factor; VEGF, vascular endothelial growth factor

Inflammation‐associated cells and molecules participate in the process of cancer cells proliferation, invasion, and metastasis directly. In normal condition, the immune cells are responsible for eliminating cancer cells. However, it may be a promoter for tumor growth in persistent inflammation conditions, which is also determined by the location and period. The direct functions of main cytokines and other special molecules in tumor development are presented in Figure 2.

##### Inflammatory factors in tumor niche

The proinflammatory factors of IL‐1 and IL‐6 from TAMs have been accepted to promote cancer cells invasion which is probably associated with the up‐regulation of their receptors. IL‐6 is considered as an antiapoptotic factor for various cancer cells.^59^ It also plays an important role in the chronic inflammation‐associated tumor development and immunosuppressive process through the Jak‐STAT3 signaling pathway. IL‐1, which includes two subtypes of IL‐1α and IL‐1β, shows different properties either in structure or cellular function. IL‐1α is suggested to induce antitumor immunity, whilst IL‐1β has been found to damage inflammatory tissue and promote tumor invasion.^86^ The role of IL‐10 in tumor growth is still controversial. On one hand, IL‐10 as well as IL‐6 were confirmed as negative regulators for innate immune cells that destroy the immunity to tumor cells. However, several other researchers have found that the high expression of IL‐10 make efforts to suppress angiogenesis and promote malignant cells apoptosis in tumor niche.^87^, ^88^ Besides, TNF‐α has also been implicated in inflammatory‐involved tumorigenesis and shows a high level in tumor cells.^89^, ^90^, ^91^ It can be produced by TAMs and other inflammatory cells as well and then promote tumor cells survival through the NF‐κB signaling pathway.^92^, ^93^ IL‐17 has also been suggested as an important member in tumor environment, especially for inflammatory‐associated disease.^94^ It is further manifested by stimulating angiogenesis in tumor tissue and then promote further tumor development.^95^

##### Chemokines in tumor niche

As is known, chemokine is the biggest subfamily of cytokines in inflammatory context. They are expressed widely by macrophages and other inflammatory cells in inflammation, and no exception in tumor microenvironment. Chemokines are divided into four types of CXC, CC, C, and CX_3_C family according to the primary structure, which usually includes CCL12, CCL13, CCL17, CCL18, CCL20, CCL22, and CXCL8.^96^ Correspondingly, CXCR, CCR, CXR, and CX_3_C are the four types of receptors to chemokine‐ligands, respectively. The effect of chemokines on tumor growth can be described directly or indirectly. On one hand, besides leukocyte, the chemokine receptors are also expressed by tumor cells on a cell‐dependent manner. As was reported, chemokines from TAMs including monocyte chemotactic protein‐1, macrophage inflammatory protein‐1α,β, macrophage derived chemokines, and IL‐8 are dedicated to tumor growth directly by interacting with their receptors.^97^, ^98^ On the other hand, chemokines from TAMs are also reported to possess the ability of attracting other cells to tumor niche, such as leukocyte and vascular endothelial cells, which make effects on tumor growth indirectly. Besides, they also give rise to the migration of cancer cells to other organs via circulation.^99^

##### Growth factors in tumor niche

Growth factors including VEGF, PDGF, EGF, TGF‐β, and FGF from TAMs are widely regarded as a potential mediator to promote the survival and proliferation of cancer cells,^100^ which can be secreted by fibroblasts. VEGF is well known to stimulate angiogenesis and then provide nutrient for tumor growth.^101^ PDGF, as the most important growth factor for pericytes, contribute to angiogenesis by stimulating pericytes.^102^ FGF has been confirmed to increase the gene expression of VEGF receptors and accelerate the proliferation of endothelial cells and fibroblasts, thus promoting angiogenesis further. Besides, it also makes contributions to the migration of cancer cells directly.^103^ EGF also contributes to the invasion and metastasis of cancer cells in the presence of other cytokines. Notably, there is a mutual regulation between these angiogenic‐related factors. For instance, TGF‐β with low dose usually supports tumor growth by strengthening the activity of angiogenic factors (such as VEGF and FGF) and proteases which contribute to angiogenesis. On contrary, TGF‐β in high level goes against angiogenesis in tumor niche by inhibiting the growth of endothelial cells.^104^, ^105^ As proposed, growth factors also modulate the tumor development by regulating other immune cells in local microenvironment. Such as TGF‐β, which regulate the comprehensive inflammatory response via affecting T‐cells behavior, thus to inhibit the tumor formation.

##### Other special conditions in tumor niche

The tumor niche is an acidic condition (pH < 7.0) which supposes to support tumor growth and metastasis. It partially ascribed to the acidic secretion from TAMs due to the fermentative metabolism of solid tumor.^106^ The acidic condition cannot just promote angiogenesis by inducing a higher expression of VEGF, but also activate the acid‐dependent proteases and accelerate ECM degradation, which is helpful to cancer cells invasion and metastasis. Aside from cancer cells, a large amount of MMP is highly expressed by TAMs, especially the MMP‐2 and MMP‐9, which are activated by chemokines.^20^ The main contributions of MMPs are promoting malignant cells invasion via degrade specific ECM, and participating angiogenesis by affecting cell adhesion. Besides, redundant ROS accumulation from leukocyte in local inflammation is another important carcinogenic factor by damaging the DNA. In many tumors, PGE2 hold a higher level than that in normal cells. It functioned as a promoter to proliferation, invasion and metastasis of cancer cells and an inhibitor to immunologic function. In a nutshell, PGE2 impairs immune activities in tumors, and most importantly also acts as supporter to malignancy at different levels.^107^, ^108^, ^109^, ^110^

#### 2.4.2 Cytokine‐involved signaling pathways in tumor

##### NF‐κB signaling pathway

NF‐κB, as one of the most important transcription factors, plays a central role in the complex network of cytokines and acts as a crucial inducer for cancer in inflammatory microenvironment.^111^, ^112^ In NF‐κB involved pathways, when cells are stimulated by physical factors (eg UV and stress) or chemical substances (eg cytokines of IL‐1 and TNF‐α), the RANK signaling pathway will be activated by the kinase and then generate a phosphorylation of IκB.^113^ Subsequently, the NF‐κB transcription factor was released and then entered into nucleus to regulate the gene expression when combined with the targeted gene promoter. Herein, it is worth noting that the NF‐κB is silent in normal cells but active in cancer cells, and can always be activated by the proinflammatory cytokines from both cancer cells and immune cells.^114^, ^115^ In current opinion, the mechanism of NF‐κB dependent tumor progression can be concluded as follows: the gene expression of VEGF and IL‐8 become abnormal and the transcription of MMPs become active in angiogenesis process, both of which will accelerate ECM degradation and the cancer cells invasion to surrounding tissue. Besides, NF‐κB can also induce the gene expression of IL‐6 that supports cancer cells survival. Indeed, the NF‐κB makes contributions to tissue remolding, tumor initiation, promotion, and metastasis processes in tumor microenvironment in a cell type dependent way. For instance, NF‐κB, has been shown to initiate tumor progression by elevating the production of reactive oxygen and nitrogen species.^54^ It is also considered as an important signals to stimulate tumor initiation by producing the mutator enzyme activation‐induced cytidine deaminase.^116^ In fact, the major effect of NF‐κB on tumorigenic is to promote cancer cells proliferation and inhibit apoptosis.^117^, ^118^

##### Jak‐STAT3 signaling pathway

In current researches, STAT3 is found to function as an oncogene and be highly activated in inflammatory‐associated cancer. The activation of STAT3 is closely related to IL‐6 and other cytokines secretion in inflammation period primarily, and also relevant to environmental stimulus like UV radiation, infection, and stress.^119^, ^120^ Briefly, STAT3, which binds to the targeted gene promoter to regulate the expression of related genes, is activated by the paracrine effect of IL‐6 and acts on the IL‐6R/gp130 receptor on target cell.^121^ IL‐10, which might promote tumor development when acting as a proinflammatory factor, rather than the antiinflammatory factor, is also involved in the Jak‐STAT3 activation.^122^ Besides, STAT3 also promotes the expression of IL‐10 with production of immunosuppressors in tumor niche. In this process, the gene expressions of antitumor cytokines like IL‐12 and IFN are inhibited by STAT3. Herein, cytokines is transformed from antitumor IL‐12 to tumorigenic IL‐23, both of which belong to the IL‐12 family of proinflammatory cytokines. In addition, PD‐L1 and PD‐L2, which were expressed in TAMs, are considered to be mediated by IL‐27 induced STAT3 signaling pathway as well.^123^ Generally, the cytokine mediated STAT3 gives rise to a high expression of genes that involved in proliferation, survival, antiapoptotic, and immune suppression of cancer cells.

##### Chemokine‐receptor dependent signaling pathway

As mentioned above, chemokines are widely expressed in inflammatory cells, including in tumor niche. Chemokines have been considered helpful to cell movement. Indeed, the combination of chemokines with their specific receptors can contribute to cell migration either in a normal condition or in a disease state. Briefly, the inflammatory cytokines from TAMs in tumor microenvironment increase the expression levels of chemokine‐receptors in malignant cells, hence strengthening the binding with their ligands. For example, TNF‐α stimuli always increases the expression of CXCR4 in malignant cells, which can promote tumor progression by direct and indirect mechanism. Then, CXCR4 functions as the specific receptor of CXCL12 to determine the extent and location of malignant cells metastases. It is also a prediction for different cancers. Moreover, malignant cells also express other chemokine receptors such as CCR and CXCR in an organ‐specific way to affect cell migration. In this regard, the migration of inflammatory cells is closely associated with tumor growth. For instance, CCR5 blockage will inhibit breast cancer cells growth, resulting a decreased monocyte recruitment into developing tumors.^124^ Besides, the inflammatory cytokines of IL‐1β and IL‐6 can also increase the expression of their corresponding chemokine‐receptors.^125^ Basically, the crosstalk between TAMs and cancer cells are mediated by the chemokine‐receptor largely, but it is not fully established and requires further investigation.

##### TGF‐β dependent signaling pathway

TGF‐β superfamily signaling pathways (TGF‐β1, TGF‐β2, and TGF‐β3) related with M2‐like TAMs also play dual roles of tumor promoter and suppressor in different stages, including tumor initiation, formation, maintenance, and progression. On one hand, TGF‐β mediated signals and the interaction between cells are confirmed to provide a favorable microenvironment for tumor metastasis and progression.^126^ It largely depends on the effect of remodeling tumor microenvironment (such as ECM, cells distribution and cytokines secretion) and regulating the interactions with other cells which express TGF‐β receptors (TGF‐BR). More and more evidences have revealed that TGF‐β mediated signaling is closely relevant with the activation of their receptors (TGF‐BR1 and TGF‐BR2) and Smad‐dependent transcription factors (Smad1/5/8, Smad2/3 and Smad4).^127^ Aside from TAMs, TGF‐β are also liberated from other immune cells, as well as cancer cells and stromal cells in tumor microenvironment. TAMs‐derived TGF‐β makes a significant influence on other immune cells which are responsible for the defense of cancer cells. For instance, the recognized potential of NK cells to cancer cells is impaired by TGF‐β via suppressing the effective molecules expression on its surface, leading to a reduced NK cell‐dependent cytolysis and clearance of tumor cells eventually. Besides, TGF‐β can also mediate the expression of other growth factors in endothelial cells and TAMs by autocrine and paracrine modes to modulate angiogenesis which account for tumor formation and progression on a dose‐dependent pattern. Moreover, TGF‐β involved signaling in tumor metastasis is cell type dependent. As previously reported, TGF‐β promotes breast cancer cells motility and metastasis by up‐regulating the gene expression of integrins.^128^, ^129^ Another example is in hepatoma carcinoma environment, TGF‐β enhanced cell migration and invasion by increasing the gene expression of chemokine receptors.^130^ In conclusion, the promotion of TGF‐β signaling on tumor development lies largely on the increased angiogenesis, decreased immune performance and more regular extra‐cellular architecture. However, TGF‐β and TGF‐β signals are also well‐known as tumor suppressors. As was mentioned previously, two types of important factors of TGF‐BR and Smad mediators are involved in TGF‐β signaling pathway. Examples are illustrated to confirm that the malignant transformation induced widely when blocking the TGF‐BR/Smad pathway.^131^ In summary, TGF‐β signaling pathways exhibit an inhibiting effect in the early phase of tumor formation through delaying cell cycle, inducing apoptosis and suppressing the gene expression of related cytokines.

**Figure 3:**
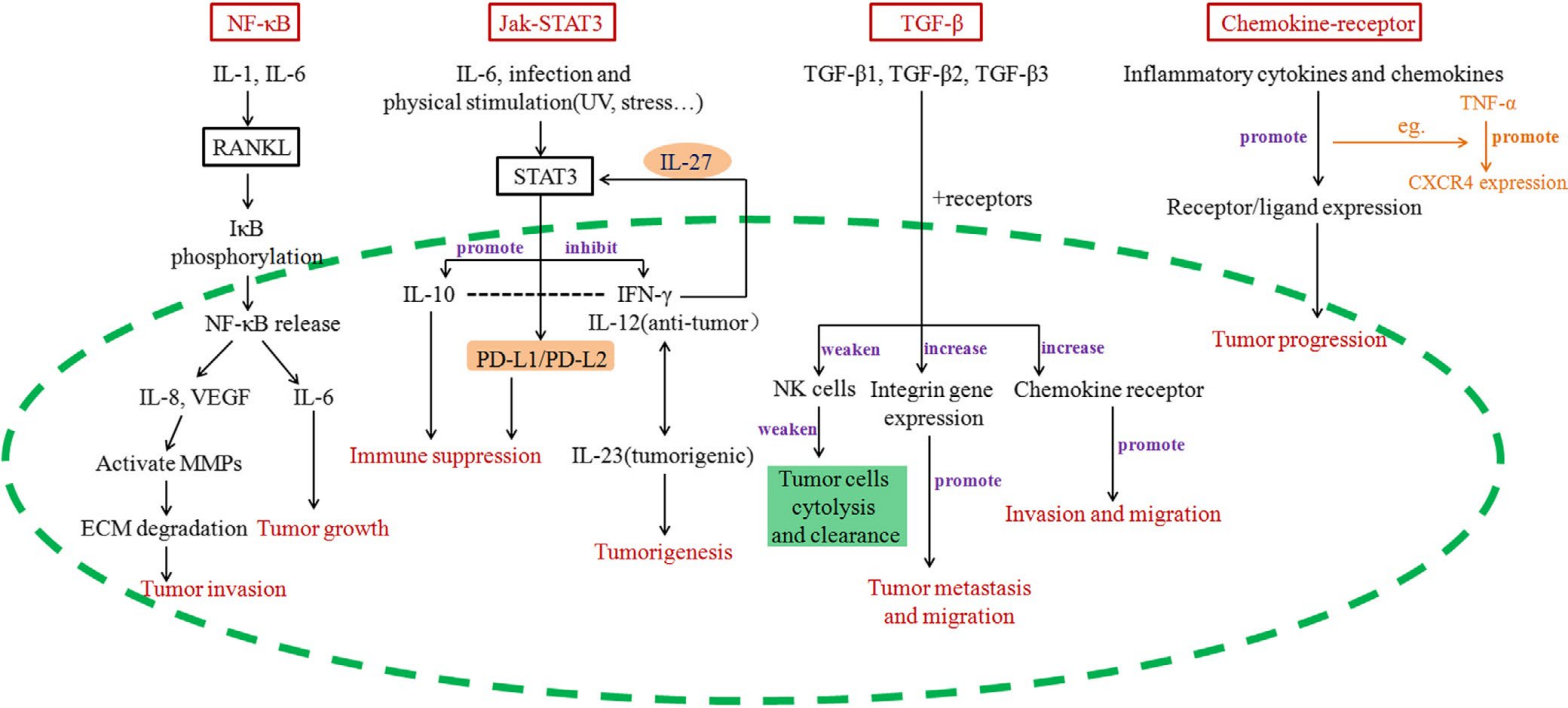
Tumor‐associated signaling pathways and their contributions. Abbreviations: ECM, extracellular matrix; IFN‐γ, interferon‐γ; IL, interleukin; MMP, matrix metalloproteinase; NF‐κB, nuclear factor‐κB; PD‐L1, programmed cell death ligand 1; STAT3, signal transduction and transcription activator 3; TGF‐β, transforming growth factor‐beta; TNF‐α, tumor necrosis factor alpha; VEGF, vascular endothelial growth factor

In fact, there is a complex correlation and interaction, rather than working independently between these signaling pathways in vivo. For example, the motility of breast cancer cells is regulated by NF‐κB signaling through an up‐regulated expression of chemokine receptor‐CXCR4. Therefore, the result is a comprehensive effect of critical processes in tumor development (such as angiogenesis, immunity and chronic inflammation) and the interaction between various cells, cytokines and diverse signaling pathways in local microenvironment. A brief summary of these signaling pathways and their roles in tumor development is shown in Figure 3.

## 3 CONCLUSION

TAMs are the most abundant inflammatory cells in tumor microenvironment. A variety of inflammatory mediators such as chemokines, growth factors, pro‐ and anti‐inflammatory cytokines and proteases from TAMs are involved. TAMs make a huge contribution to tumor development by modulating angiogenesis, ECM remolding, chronic inflammation and immune suppression processes via complex interaction between signaling pathways of NF‐κB, Jak‐STAT3, chemokine‐receptor interaction and TGF‐β associated. However, it is worth noting that the TAMs are different from the original macrophages in normal physiological microenvironment in which TAMs are the production of reprogrammed macrophages by the tumor microenvironment, such as tumor‐derived exosomes, cytokines, and other immune cells. Indeed, the reprogrammed modulation is bilaterally which results in a different cytokines secretion profile of TAMs. Therefore, the interactions between macrophages and other cells in tumor microenvironment should be altered as well to create a new niche to support cancer cells survival and development.

## CONFLICT OF INTEREST

None declared.
